# (+)-Vitisin A Inhibits Osteoclast Differentiation by Preventing TRAF6 Ubiquitination and TRAF6-TAK1 Formation to Suppress NFATc1 Activation

**DOI:** 10.1371/journal.pone.0089159

**Published:** 2014-02-18

**Authors:** Wen-Fei Chiou, Yu-Ling Huang, Yen-Wenn Liu

**Affiliations:** 1 National Research Institute of Chinese Medicine, Ministry of Health and Welfare, Taipei, Taiwan, ROC; 2 Department of Biotechnology, Hungkuang University, Taichung, Taiwan, ROC; 3 Department of Cosmetic Science, Chang Gung University of Science and Technology, Taoyuan, Taiwan, ROC; University of Kansas School of Medicine, United States of America

## Abstract

We recently reported that oral administration of a (+)-vitisin A-enriched product prepared from *Vitis thunbergii* obviously ameliorated bone loss in ovariectomized mice and (+)-vitisin A was able to inhibit receptor activator of NF-κB ligand (RANKL)-induced osteoclast differentiation in RAW264.7 cells. Here we further clarified the mechanism(s) by which (+)-vitisin A targets osteoclastic differentiation and activity. Osteoclast-characteristic enzyme activity was determined using gel zymography or spectroflurometric-based assay. Expression of signal molecules was analyzed via Western blot or immunoprecipitation. Results showed that (+)-vitisin A suppressed RANKL-induced multinuclear cells (MNCs) formation and bone resorption which was accompanied with reduction in β3 integrin, osteoclast stimulatory transmembrane protein (OC-STAMP), matrix metalloproteinase-9 (MMP-9) and cathepsin K proteins expression. (+)-Vitisin A also down-regulated the proteolytic activities of MMP-9 and cathepsin K via targeting at the late stage function. (+)-Vitisin A prominently abrogated RANKL-triggered nuclear translocations of NF-κB, AP-1 (c-Fos/c-Jun dimer) and associated induction and nuclear accumulation of nuclear factor of activated T cells c1 (NFATc1). The upstream IκB degradation as well as ERK and JNK phosphorylation were also substantially repressed. Transfection with siRNA targeting tumor necrosis factor receptor associated factor 6 (TRAF6) clearly restrained RANKL-induced MNCs formation and NFATc1 induction. Interesting, RANKL triggered poly-ubiquitination of TRAF6 and associated TRAF6-TAK1 (transforming growth factor β-activated kinase 1) complex formation was prominently attenuated by (+)-vitisin A. Furthermore, the interaction between c-src tyrosine kinase (c-Src) and β3 was markedly induced by RANKL stimulation. (+)-Vitisin A significantly attenuated this interaction when concomitant treated with RANKL in RAW264.7 cells, but failed to affect c-Src/β3 complex formation when post-cultured with MNCs. Taken together, (+)-vitisin A suppressed bone resorption possibly via interruption of RANKL-induced TRAF6 ubiquitination and associated downstream signaling pathways. Furthermore, action through negative regulation of the proteolytic activity of MMP-9 and cathepsin K might also contribute to the anti-resorption effect of (+)-vitisin A.

## Introduction

(+)-Vitisin A is a dominant stilbene contained in the roots of *Vitis thunbergii* Sieb. & Zucc. (*Vitis ficifolia* Bge. Vitaceae), a well-known herbal remedy traditionally used in Taiwan for diarrhea, jaundice, hepatitis, fracture and injury [1]. Accumulating use experience suggests an alcoholic drench of the roots of *V. thunbergii* also helps cure bone fractures, contusions and prevents bone loss, suggesting that this herb might either stimulate new bone formation or inhibit bone resorption. We have reported that orally administered with (+)-vitisin A-enriched preparation for eight weeks significantly ameliorated the deterioration of bone mineral density and reversed all the ovariectomy-induced changes in microarchitecture when starting post-treatment at two weeks after ovariectomy [2]. How (+)-vitisin A displayed beneficial effect on preventing bone loss is unknown? Increasing evidence indicates that osteoclasts play crucial role in bone loss. Osteoclasts are derived from monocyte/macrophage lineage precursor cells through a differentiation process primarily governed by two key cytokines, macrophage colony-stimulating factor (M-CSF) and receptor activator of nuclear factor kappa B ligand (RANKL). RANKL provides an essential signal for osteoclastic differentiation while M-CSF supports cell proliferation and survival during osteoclastogenesis.

The cytoplasmic domain of RANK interacts with members of the family of tumor necrosis factor receptor-associated factors (TRAFs) that mediate activation of the inhibitor of κB (IκB) kinase (IKK) and mitogen-activated protein kinases (MAPKs) [3]. Among these TRAF molecules, TRAF6 has been shown to be a pivotal component in the RANK signaling pathway [4]. The N-terminal RING domain of TRAF6 belongs to a growing family of ubiquitin ligases and is critical for TRAF6 auto-ubiquitination [5], [6]. RANKL-triggered ubiquitination of TRAF6 initiates the activation of IKK and MAPKs signal cascades. Transforming growth factor β-activated kinase-1 (TAK1) is a member of the MAPK kinase kinase (MAPKKK) family [7] that mediates IKK and MAPKs activation via interaction with TRAF6. Mizukami et al. [8] had found that RANKL stimulation not only activates endogenous TAK1 but also forms the TRAF6-TAK1 complex associated with RANK. Additionally, activated RANK prompts two distinct signaling pathways: one promotes osteoclast formation through signaling pathway as above described, and the other, in which the c-src tyrosine kinase (c-Src) and αvβ3 integrin are required, organizes the cell's cytoskeleton and stimulates its resorptive capacity [9]. Src is activated in osteoclasts after the cells bind to integrins in bone matrix during the initial stages of bone resorption [10]. This initiates signaling that leads to the complex intracellular cytoskeletal reorganization that is required for polarization of the cytoplasm and ruffled border formation.

Certain transcription factors are involved in regulating the expression of genes that characterize osteoclasts [10]. For example, RANKL stimulates activator protein 1 (AP-1) through MAPK activation and NF-κB through IKK activation. Furthermore, the nuclear factor of activated T cells c1 (NFATc1), a member of the NFAT family of transcription factors, is important for RANKL-induced osteoclastic differentiation. Genetic studies also showed that deficiency in those transcription factors can block osteoclastogenesis [11]–[13].

Osteoclast surface attachment is a first and essential step in a cascade of events that continues with osteoclast polarization, formation of podosomes and a sealing zone, and finally secretion of acids and proteases for the resorption of bone. Adhesive interaction between cells and extracellular matrix proteins is mediated by cell surface receptor integrins (αvβ3, αvβ5, α2β1, and αvβ1) [14], [15]. Fusion-mediated giant cell formation is critical for osteoclast maturation and bone resorption [16]. Recently, osteoclast stimulatory transmembrane protein (OC-STAMP) has been identified as the RANKL-induced protein that promotes multinucleated osteoclast formation since treatment of its specific antibody inhibited the formation and bone resorptive activity of mature osteoclasts [17]. Proteinases also play an important role in bone physiology, including the solubilization of bone matrix [18]. Cysteine proteinases (such as cathepsins) and matrix metalloproteinases (MMPs) have been identified as the main proteinases active in these processes [19]. Among MMPs, MMP-9 is the most abundant gelatinolytic MMP in osteoclasts and is the main MMP involved in the invasive activity of osteoclasts [20]. Cathepsin K, a 24 kDa lysosomal cysteine proteinase, is expressed at high levels in osteoclasts and plays a key role in bone resorption by degradation of type I collagen [21].

We recently found that (+)-vitisin A not only significantly repressed RANKL and M-CSF induced osteoclast differentiation in cultured bone marrow derived macrophages [2], but also markedly inhibited such process in cultured RAW264.7 macrophages stimulated with RANKL alone and without effect on cell viability. RAW264.7 cell is a well-established system for studying osteoclastogenesis since differentiation and maturation process can be easily and successfully accomplished without M-CSF. This provides a more suitable model to investigate RANKL-related signaling pathway compared to bone marrow macrophages which require both mCSF and RANKL for stimulating osteoclastic differentiation. Other advantages of using RAW264.7 cells include easy accessibility, high prolific ability (leukemic cells), and amenable to genetic manipulations (transfection studies) [22]. Therefore, how (+)-vitisin A impeded RANKL-induced osteoclastogenesis was studied by using RAW264.7 cells to explore the precise mechanisms. The function of osteoclasts was monitored by bone resorption and proteolytic activity assay. Western blot and immunoprecipitation were used to analyze osteoclast-associated proteins expression and signaling pathways.

## Materials and Methods

### Chemicals and reagents

(+)-Vitisin A (CAS NO. 142449-89-6) was isolated from the roots of *Vitis thunbergii* by the authors. This herb was purchased in Taipei, Taiwan, in July 1998 and identified by Mr. Jun-Chih Ou, a taxonomist retiring from our institute. A voucher specimen (NRICM-98-010) is deposited at the Herbarium of National Research Institute of Chinese Medicine, Republic of China. In brief, the dried roots of *Vitis thunbergii* (3 kg) were extracted in ethanol. After a series of separation and individual chromatography on silica gel column eluting with CH_2_Cl_2_−MeOH (10∶1) and on Sephadex LH-20 column eluting with MeOH−H_2_O (3∶1), 5 stilbenes including (+)-vitisin A were obtained. More detailed information for the HPLC analysis and the structure identification of (+)-vitisin A (purity 99.4%) was described in our previous work [23], [24]. Dulbecco's Modified Eagle Media (DMEM) and fetal bovine serum (FBS) were purchased from Gibco BRL (Grand Island, NY, USA). Mouse RANKL was purchased from R&D Systems (Minneapolis, MN, USA); 3-[4,5-dimetylthiazol-2-yl]-2,5-diphenyltetrazolium bromide (MTT) and Leukocyte acid phosphatase (TRAP) kit were purchased from Sigma Chemical Co. (St. Louis, MO, USA); Bovine bone slices were purchased from Nordic Biosciense (Herlev, Denmark). Antibodies against total or phosphorylated IKKβ, ERK, and JNK, antibodies against c-Fos, c-Jun, NFATc1, β3 integrin, c-Src, OC-STAMP and MMP-9 were all purchased from Cell Signaling Technology (Beverly, MA, USA). Antibodies against cathepsin K, IκB, NF-κB p65, TRAF-6, TAK-1, β-actin, histone, horseradish peroxidase-labeled goat anti-rabbit IgG and goat anti-mouse IgG were from Santa Cruz Biotechnology Inc. (Santa Cruz, CA, USA). Ubiquitin monoclonal antibody was purchased from Abcam company (Cambridge, MA, USA). Enhanced chemiluminescence (ECL) Western blotting detection reagent was from GE Healthcare (Little Chalfont, Buckinghamshire, UK). ECL plus kit was from Amersham Biosciences Inc. (Piscataway, NJ, USA). All other chemicals and reagents were from Sigma-Aldrich. (St. Louis, MO, USA). (+)-Vitisin A was dissolved in dimethyl sulfoxide (DMSO) at 0.1 M as a stock solution and diluted with culture medium. A final DMSO concentration in the culture was less than 0.1% and did not show observably artificial or cytotoxic effect. The concentrations used for (+)-vitisin A were between 1 and 20 µM.

### Osteoclast differentiation assay

The culture condition for RAW264.7 macrophage (American Type Culture Collection, Manassas, VA, USA) was described previously [25]. For differentiation, cells were cultured for 4–5 days in DMEM containing 100 ng/ml recombinant murine RANKL (defined as differentiation medium) in the absence or presence of (+)-vitisin A (1, 5, 10, 15, and 20 µM). Osteoclast differentiation was analyzed by measuring tartrate-resistant acid phosphatase (TRAP) activity and counting the number of TRAP-positive multinucleated cells (TRAP^+^ MNCs) as described below.

### TRAP activity measurement and TRAP-positive multinucleated cells (TRAP^+^ MNCs) staining

After differentiation, osteoclast was lysed and incubated with a reaction buffer containing *p*-nitrophenylphosphate (*p*NPP) to measure the TRAP activity as described previously [25]. TRAP activity measured in the presence of RANKL alone was defined as control (100%). All results were expressed as relative to control. To preclude the possibility that the attenuation in TRAP activity was due to cytotoxicity, cell viability was simultaneously measured by MTT assay. The cells cultivated in differentiation medium alone served as the control (100%). To confirm the generation of TRAP^+^ MNCs, cells were fixed with 3.7% formalin, permeablized with 0.1% Triton X-100, and finally stained for TRAP with the Leukocyte Acid Phosphatase Kit (Sigma, Cat. No. 387A-1KT). TRAP^+^ MNCs containing five or more nuclei were counted under the light microscope [25].

### Functional bone resorption assay

The resorptive function of mature MNC derived from RANKL-differentiated RAW264.7 cells was analysed on Osteologic Plates (BD Biosciences, San Jose, CA, USA). Briefly, cells were cultured in differentiation medium with or without (+)-vitisin A (5, 10, and 15 µM) for 7–8 days. Cells were then removed in 1 N NaOH for 20 min, and the slices were washed twice with PBS and the resorption pits were visualized by stained with Mayer's hematoxylin (Sigma) for 30 s. The resorption area was observed under a light microscope and analyzed by Image-Pro Plus^™^ [25].

### Measurement of matrix metalloproteinase activities

For assay of MMP-9 activity, zymography was performed as described using gelatin gels (Bio-Rad, Hercules, CA, USA) [25], [26]. After culture with RANKL for 5 days in the absence or presence of (+)-vitisin A (5, 10, and 15 µM) respectively, the supernatant was assessed for gelatinolytic activity and the protein concentration was measured by Bio-Rad DC Protein Assay Kit (Bio-Rad). As the protein levels were not modulated by RANKL and (+)-vitisin A treatment, thus equal volumes of culture supernatants were used for the assays. All the samples were electrophoresed on 7.5% sodium dodecyl sulfate-polyacrylamide gel (SDS-PAGE) copolymerized with 0.1% gelatin. Gels were washed for 1 hour in 20 mM Tris HCl, pH 7.8, and 2.5% Triton X-100 and then incubated in 20 mM Tris HCl, pH 7.8, 1% Triton X-100, 10 mM CaCl_2_, and 5 µM ZnCl_2_ at 37°C overnight. Gels were stained with Coomassie blue R-250, the appearance of fainter bands as a result of gelatin degradation due to in situ MMP activity. In another set of experiments, (+)-vitisin A or PBS was post-added to the osteoclasts culture that had been differentiated for entire 5 days then incubated for further 3 days. After that, the supernatant was used for MMP-9 activity assay [25].

### Cathepsin K activity assay

Quenched fluorescent resonance energy transfer (QFRET) technology was used to measure the cathepsin K-inhibitory effect of (+)-vitisin A in cleaving synthetic peptide Z-Phe-Arg-AMC (Z-Phe-Arg 7-amido-4-methylcoumarinhydrochloride; SIGMA, France). After generation of mature osteoclasts, (+)-vitisin A (5, 10, and 15 µM) or E-64 (a cathepsin K inhibitor) was added to the culture for further 3 days and supernatant was harvested for fluorometric activity assay. The reaction was carried out at 37°C in 100 mM sodium acetate, 20 mM L-cysteine and 5 mM EDTA buffer (pH 5.5) in the presence of cell lysate at the concentration of 0.5 µg/ml. Fluorescence intensity was monitored by a Schimadzu RF-1501 spectrofluorimeter (Schimadzu, Japan) with excitation wavelength at 365 nm and emission wavelength at 440 nm [27], [28].

### Western blot and immunoprecipitation assay

Total cell lysate, cytoplasmic protein and nuclear fraction were prepared as described previously [29]. Harvested proteins were separated by SDS-PAGE and transferred to a polyvinylidene difluoride membrane (Amersham Bioscience, Piscataway, NJ, USA). After blocking, the membrane was incubated with primary antibodies overnight at 4°C. Secondary antibody conjugated with horseradish peroxidase was used to allow detection by the ECL or ECL plus kit and exposed to X-ray film. The film was then scanned using Epson Expression IT8 with SilverFast Ai Launcher. The images were analyzed using Fuji Multi Gauge (v2.2).

For immunoprecipitation assay, RAW264.7 cells plated in 10 cm diameter dishes were stimulated with RANKL in the absence and presence (+)-vitisin A (5, 10, and 15 µM), respectively. Cells were lysed in lysis buffer as described previously [29]. After centrifugation (10,000×*g* for 10 min at 4°C), cell extracts were immunoprecipitated with anti-TAK1 or anti-Src antibody. Co-precipitated TRAF6 or β3 integrin was detected by immunoblotting with anti-TRAF6 or anti- Src antibody. The amounts of TAK1 or c-Src in each immune complex were determined by immunoblotting using TAK1 or c-Src antibody. Band signals were visualized by the ECL system [29]. The ubiquitination of TRAF6 was also detected using an immunoprecipitation assay. In brief, cell extracts were immunoprecipitated with antibody for TRAF6. The ubiquitination of TRAF6 was detected by immunoblotting with ubiquitin antibody. The bands were visualized using film exposure with ECL substrate.

### Transfection of TRAF6 siRNA

To test the role of TRAF6 in RANKL-induced osteoclastogenesis, RAW264.7 cells were transfected with a pool of 4 specific siRNAs (Thermo Scientific, Dharmacon, CO, USA) or nonspecific control siRNA, and then treated with RANKL. The ON-TARGET plus SMART pool TRAF6 siRNA target sequences were GCACAGCAGUGUAACGGGA, GGACAAGGUUGCCGAAAUG, GAGAACAGAUGCCUAAUCA, and GCUCAAUCGUUUAAUAAGA. Briefly, RAW264.7 macrophages were transfected at 70% confluence with a final concentration of 100 nM SMART pool siRNA or nonspecific control pool using DharmaFECT-1 siRNA transfection reagents (Dharmacon) according to the manufacturer's instructions. After 24 h, the medium was replaced with differentiation medium and cells were incubated for an additional 3 days then the expression of TRAF6 was detected with anti-TRAF6 antibody. In another set of experiment, formation of MNCs and bone resorption were measurement after siRNA transfection.

### Statistical analysis

Data were expressed as mean ± SEM. All experiments were performed at least three times independently to confirm our results. The data were analyzed by one-way analysis of variance (ANOVA) followed by *post-hoc* Dunnett's t-test for multiple comparisons. A *P* value <0.05 was considered significant.

## Results

### (+)-Vitisin A represses RANKL-induced osteoclast formation and bone resorption

**Figure 1:**
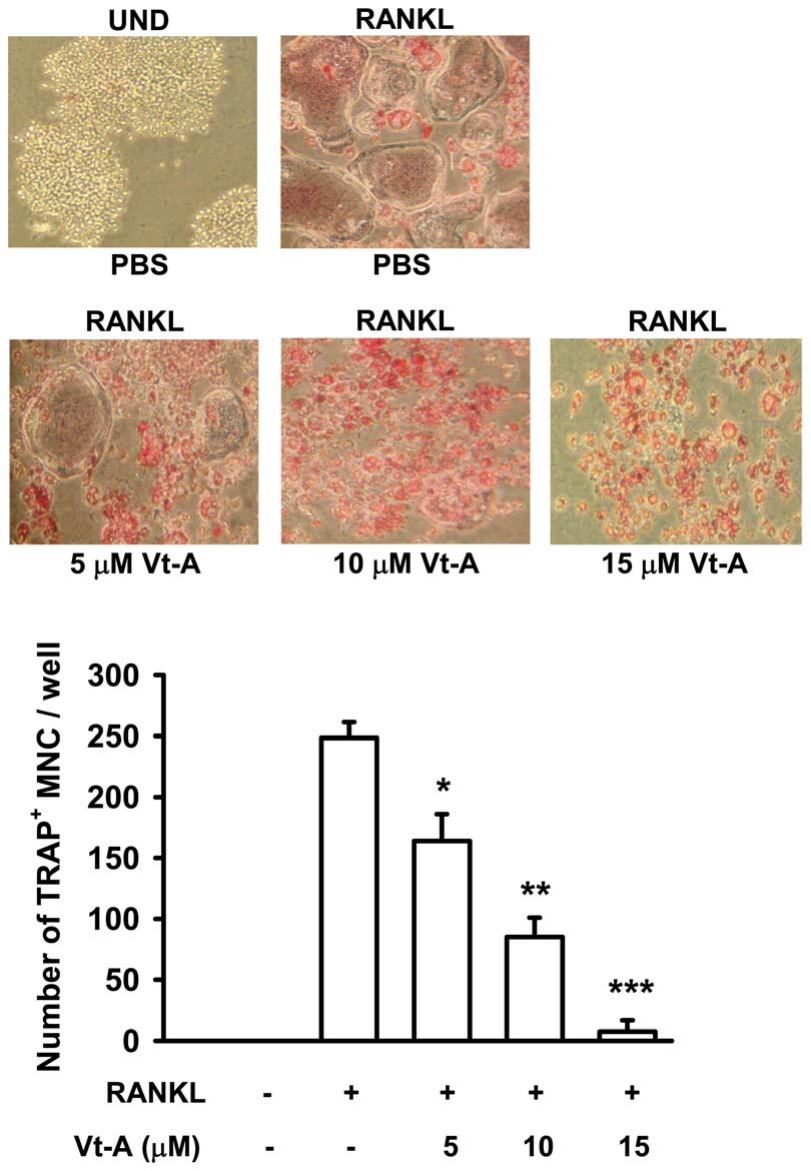
(+)-Vitisin A (Vt-A) inhibited RANKL-induced formation of tartrate-resistance acid phosphatase (TRAP)-positive multinucleated cells (MNCs) in RAW264.7 cell cultures. Cells were cultured with indicated concentrations of Vt-A in the presence of RANKL (100 ng/ml) as described in materials and methods. Summary values are shown in the bar graph and each value is the mean ± SEM of four independent experiments. *p<0.05 and **p<0.01, different from values after stimulated with RANKL alone.

**Figure 2:**
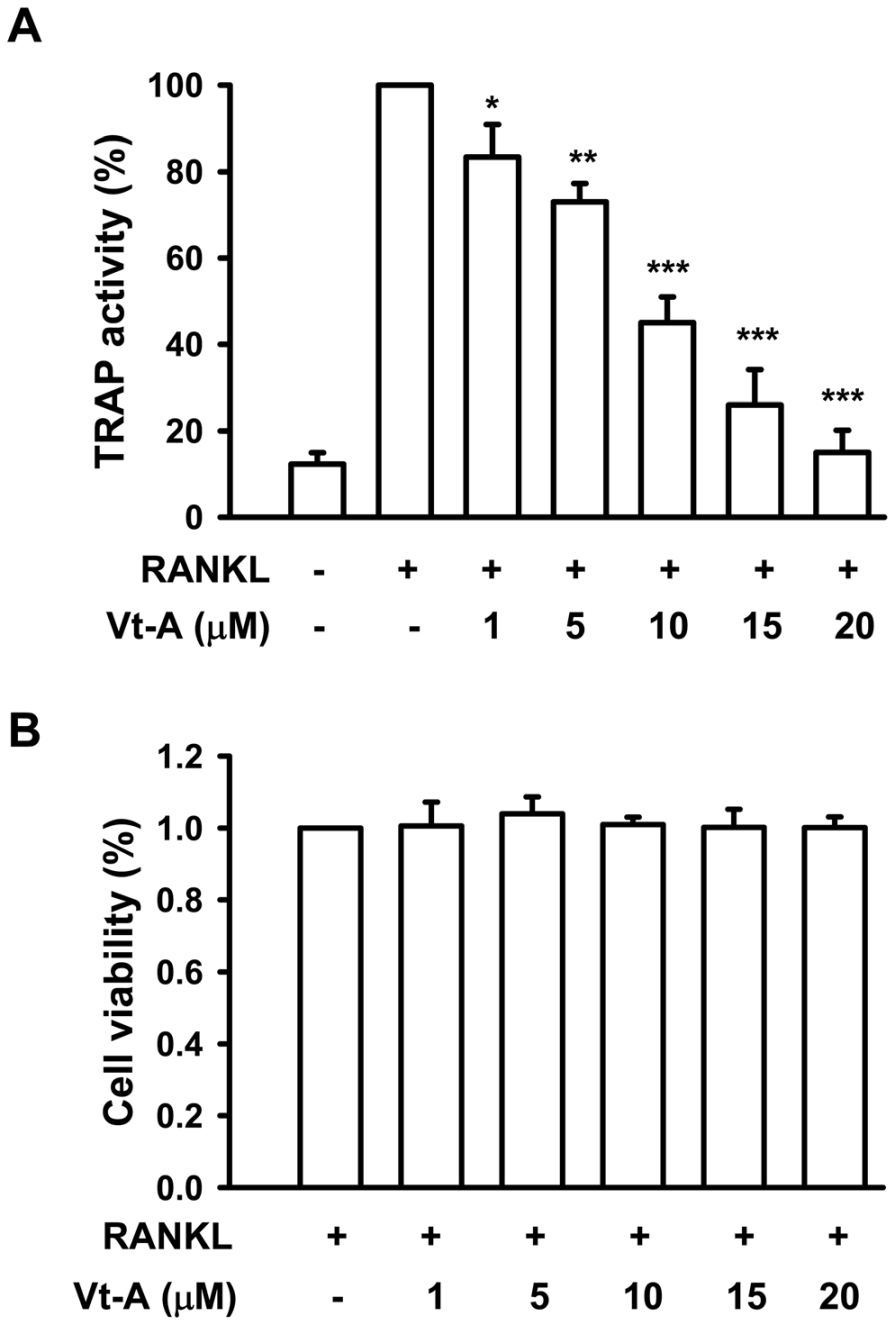
Effects of (+)-vitisin A (Vt-A) on RANKL-induced tartrate-resistance acid phosphatase (TRAP) activity and cell viability in RAW264.7 cells. (A) For osteoclast differentiation, RAW264.7 cells were stimulated with RANKL (100 ng/ml) and TRAP activity was assayed in cell lysates as described in methods. (B) The effect of Vt-A on cell viability was evaluated by MTT assay. Each value is the mean ±SEM of five independent experiments each performed in triplicate. *p<0.05 and **p<0.01, different from values after treatment with RANKL alone.

Signaling by RANKL is crucial for terminal differentiation of macrophages into osteoclasts. In undifferentiated cells (UND), there was no detectable TRAP staining observed, but RANKL produced numerous huge and TRAP-positive multinucleated cells (TRAP^+^ MNCs ≧ five nuclei). When (+)-vitisin A (5, 10 and 15 µM) was added together with RANKL, the number of TRAP^+^ MNCs was diminished concentration-dependently. Especially in the presence of 15 µM (+)-vitisin A, RANKL-induced fusion of MNCs was almost abolished (Figure 1). We also examined the inhibitory effect of (+)-vitisin A on TRAP activity. Figure 2A revealed that RANKL obviously fostered TRAP activity in RAW264.7 macrophages and this activity was dose-dependently declined in the presence of (+)-vitisin A. Cell viability was not affected by treating 1-20 µM (+)-vitisin A (Figure 2B), suggesting that ≦ 20 µM (+)-vitisin A was not cytotoxic.

**Figure 3:**
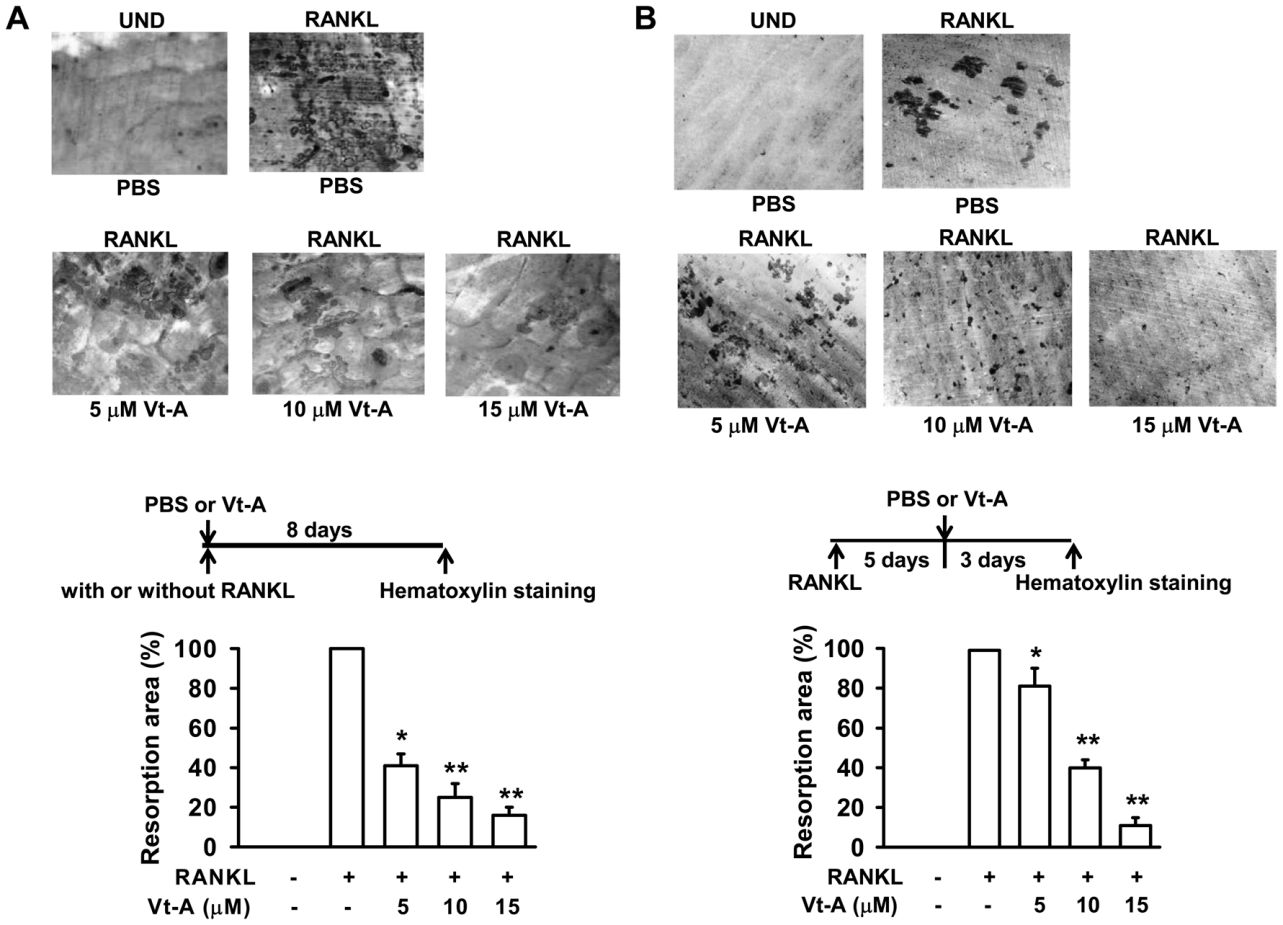
Effect of (+)-vitisin A (Vt-A) on pit-forming activity in RANKL stimulated RAW264.7 cells. (A) Cells cultured on bone slices were stimulated with RANKL for 8 days by concurrent addition of Vt-A. (B) Vt-A was added at 5 days after RANKL stimulation and differentiated for further 3 days. After removing the cells, the slices were stained with Mayer's haematoxylin to identify resorption pits. The resorption area was observed under a light microscope and analyzed by Image-Pro Plus^™^. Data represent the mean ± SEM of three to five independent experiments. *p<0.05 and **p <0.01, different from values after treatment with RANKL alone.

To determine if this suppression of TRAP^+^ MNCs formation affected the resorptive ability, RAW264.7 cells were cultured on bone slices, and then differentiated to mature osteoclasts by RANKL for 8 days in the absence or presence of (+)-vitisin A. When compared to the un-differentiated (UND) group, many resorption pits (discriminated from the gray background by an intensively dark stain) were formed on the bone slices after cultured with RANKL. As shown in Figure 3A, RANKL augmented the number and size of bone resorption areas in osteoclasts transformed from macrophages. (+)-Vitisin A strongly reduced the resorption area when added at an earlier stage of differentiation. It is surprising that (+)-vitisin A added at several days after differentiation still significantly mitigated bone resorption (Figure 3B). In particular, adding a higher concentration of (+)-vitisin A (15 µM) at late stage of differentiation also decreased the pit area, similar to treatment at earlier stage. This inhibition was not caused by cell death since (+)-vitisin A has no significant effect on mature osteoclast viability (data not shown).

### (+)-Vitisin A inhibits the proteins expression involved in osteoclast maturation and bone resorption

**Figure 4:**
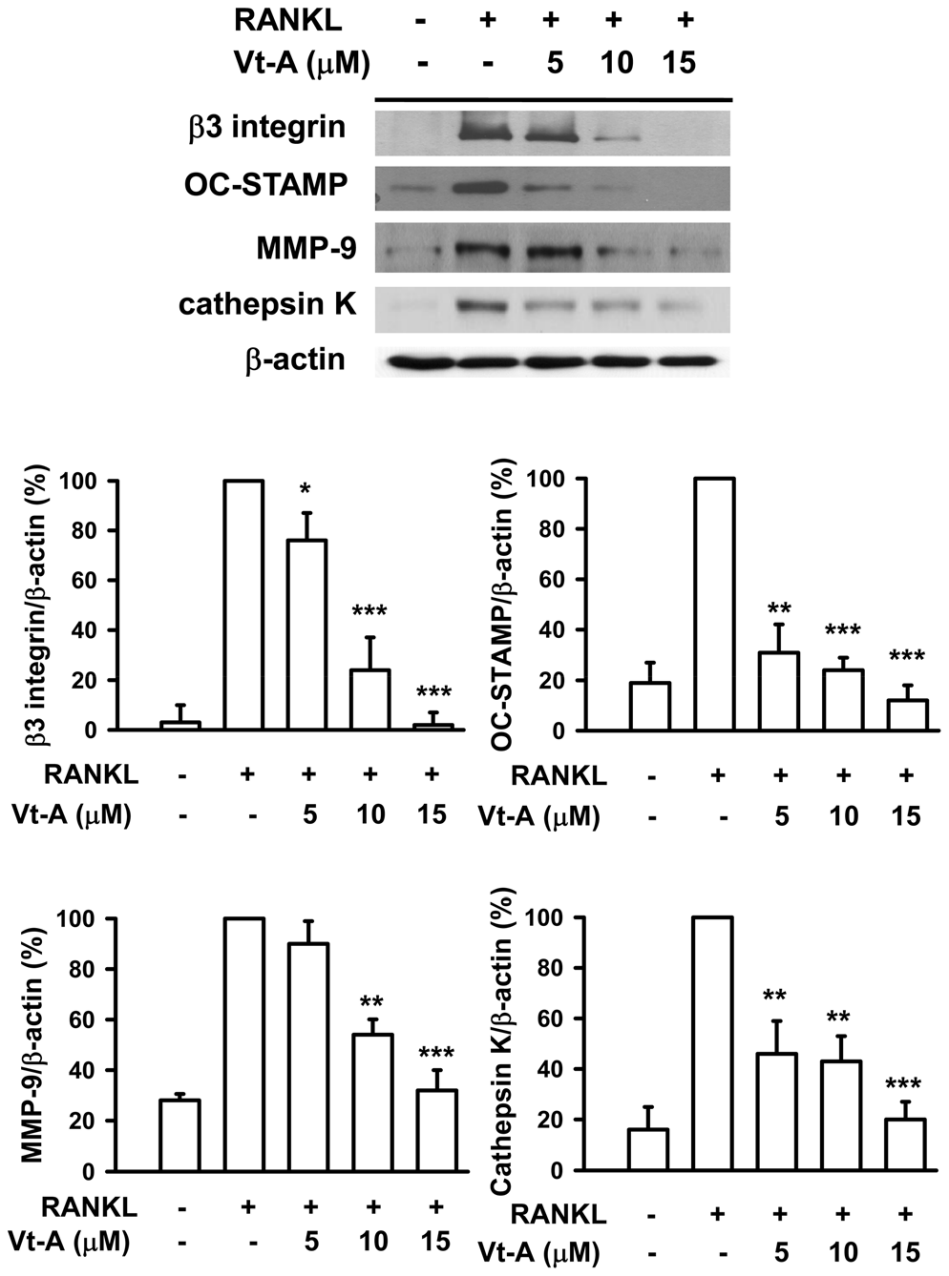
(+)-Vitisin A (Vt-A) suppressed RANKL-induced expression of β3 integrin, OC-STAMP, MMP-9 and cathepsin K in RAW264.7 cells. The relative protein levels were measured by western blot and normalized to β-actin. Data represent the mean ± SEM of at least three independent experiments. *p<0.05, **p<0.01 and ***p< 0.001, different from values after stimulated with RANKL alone.

It is known that β3 integrin plays a role in regulation of cell migration and maintenance of the sealing zone required for effective osteoclastic bone resorption [30], [31]. After RANKL stimulation for 5 days, the protein expression of β3 integrin was significantly up-regulated (Figure 4). However, the induction of β3 integrin was attenuated by addition of (+)-vitisin A concentration-dependently. OC-STAMP protein expression was increased fivefold in RANKL-stimulated cells. In the presence of (+)-vitisin A, the induction of OC-STAMP protein was reduced by at least 86% by 5 µM (+)-vitisin A and almost completely abolished by 10 µM (+)-vitisin A treatment. MMP-9 is responsible for the bone resorption mediated by mature osteoclasts [20], [32]. Compared with un-differentiated cells, MMP-9 protein expression increased approximately threefold in cells stimulated with RANKL. The addition of (+)-vitisin A decreased the induction of MMP-9 by RANKL. As shown in Figure 4, 5 µM of (+)-vitisin A had slight effect on MMP-9 expression, with up to 95% reduction after 15 µM (+)-vitisin A treatment. On the other hand, osteoclast-specific cathepsin K is elevated during maturation. Cathepsin K is secreted in a sealed zone beneath the ruffled border of the osteoclast and plays a pivotal role in bone resorption [33]. Results shown in Figure 4 also demonstrate that RANKL enhanced cathepsin K expression in RAW264.7 macrophages and such responsiveness was significantly attenuated by (+)-vitisin A.

**Figure 5:**
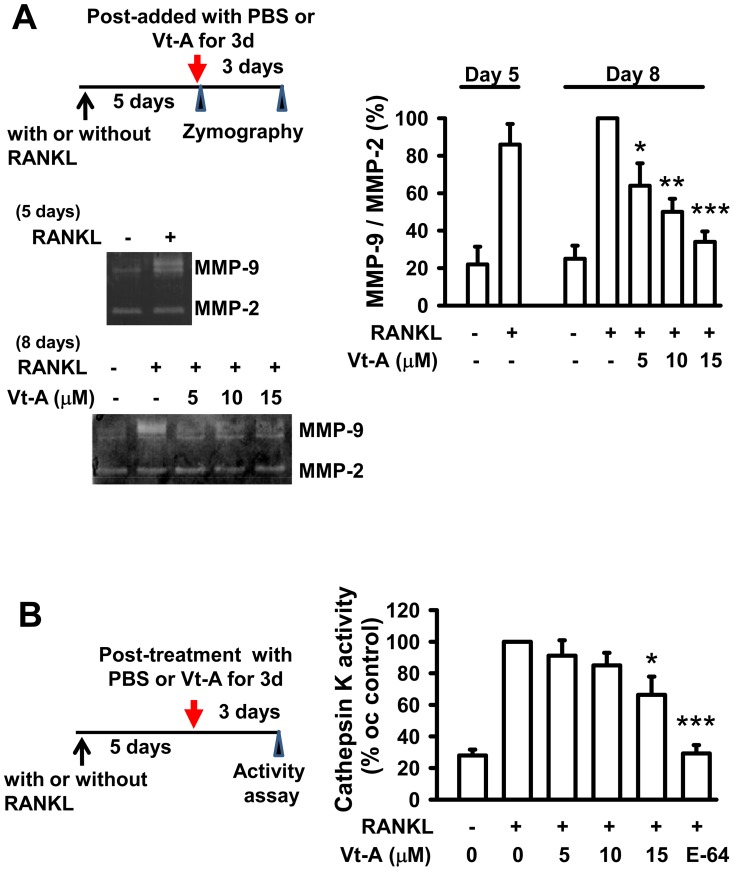
Enzyme activities of MMP-9 and cathepsin K were repressed by subsequent addition of (+)-vitisin A (Vt-A). MMP-9 activities (A) were analyzed using gelatin zymographic assays and cathepsin K activity (B) was measured by using QFRET Technology as described in methods. Data represent the mean ± SEM of four independent experiments.*p<0.05, **p<0.01 and ***p< 0.001, different from values after stimulated with RANKL alone.

### (+)-Vitisin-A down-regulates the proteolytic activities of MMP-9 and cathepsin K in mature osteoclasts

In another set of experiments, (+)-vitisin A was added to mature MNCs culture at 5 days after RANKL stimulation. After incubation with or without (+)-vitisin A for 3 more days, the supernatant was used to perform gelatin zymography to test whether or not (+)-vitisin A restricted the secreted MMP-9 enzyme activity. Compare to un-differentiated cells, MMP-9 activity was significantly increased by RANKL stimulation for 5 days evidenced by the enhancement of an unstained band at 92 kDa (Figure 5A), indicating the pro-MMP-9 activity was enhanced, Cell differentiation for the entire 8 days did not further enhance the proteolytic activity significantly. The gelatinolytic activity of MMP-9 secreted by the mature MNCs was concentration-dependently repressed by post-addition of (+)-vitisin A, suggesting down-regulation of MMP-9 proteolytic activity might also contribute to the anti-resorption effect of (+)-vitisin A. Conversely, gelatin zymography indicated the latent 72 kDa pro-form of MMP-2 is not affected by RANKL activation or by (+)-vitisin A treatment.

The effect of (+)-vitisin A on cathepsin K activity was also evaluated. After generation of resorptive osteoclasts by stimulation with RANKL for 5 days, (+)-vitisin A or E-64 (a cathepsin K inhibitor) was added to the culture for 3 more days and the supernatant was harvested for fluorometric activity assay. As shown in Figure 5B, mature MNCs (stimulated with RANKL for entire 8 days) had measurable cathepsin K enzymatic activity (about 3 folds of the undifferentiated group). Proteolytic activity of RANKL-differentiated MNCs was confirmed to be cathepsin K specific, because the addition of a selective cathepsin K inhibitor E-64 reduced cathepsin K activity by nearly 99% (Figure 5B). When (+)-vitisin A was post-added to the mature MNCs, cathepsin K activity was gradually decreased.

### Inhibition of NFATc1 auto-amplification by (+)-vitisin A

**Figure 6:**
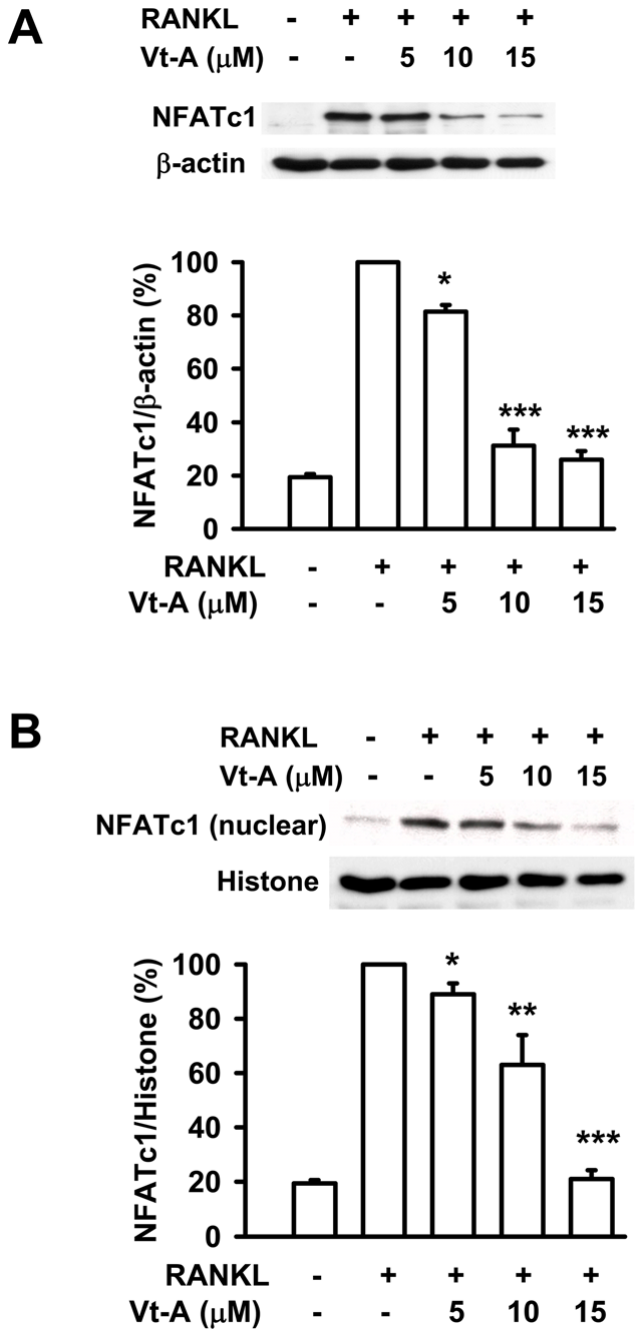
RANKL-stimulated NFATc1 induction and translocation. The (A) induction and (B) nuclear translocation of NFATc1 by RANKL stimulation were suppressed by (+)-vitisin A (Vt-A). RAW264.7 macrophages were stimulated with RANKL (100 ng/mL) for 48 h in the absence or presence of Vt-A then the total and nuclear protein were extracted as described in methods. Results are expressed as the mean ± SEM for each group from four to five separate experiments normalized for histone (for nuclear protein normalization) or β-actin (for total protein normalization) respectively. *p< 0.05, **p< 0.01 and ***p< 0.001, different from values after stimulated with RANKL alone.

To delineate the mechanism by which (+)-vitisin A inhibited osteoclastogenesis, we initially investigated its effect on the induction of NFATc1. As compared to the un-differentiated group, NFATc1 protein was potently induced by RANKL stimulation by approximately 5-fold. The induction of NFATc1 was down-regulated by co-treatment with (+)-vitisin A (Figure 6A). Next, we examined the nuclear localization of NFATc1 in (+)-vitisin A-treated cells after RANKL stimulation. Figure 6B shows that RANKL strongly elevated the nuclear localization of NFATc1 in addition to its expression. (+)-Vitisin A treatment substantially decreased NFATc1 level in the nuclear fraction of RANKL-stimulated cells. These observations suggested that (+)-vitisin A might disengage the NFATc1 auto-amplification loop by interfering with the nuclear translocation of NFATc1 in RAW264.7 cells, reducing further expression of NFATc1.

### (+)-Vitisin A suppresses RANKL-induced activation of AP-1 and NF-κB

**Figure 7:**
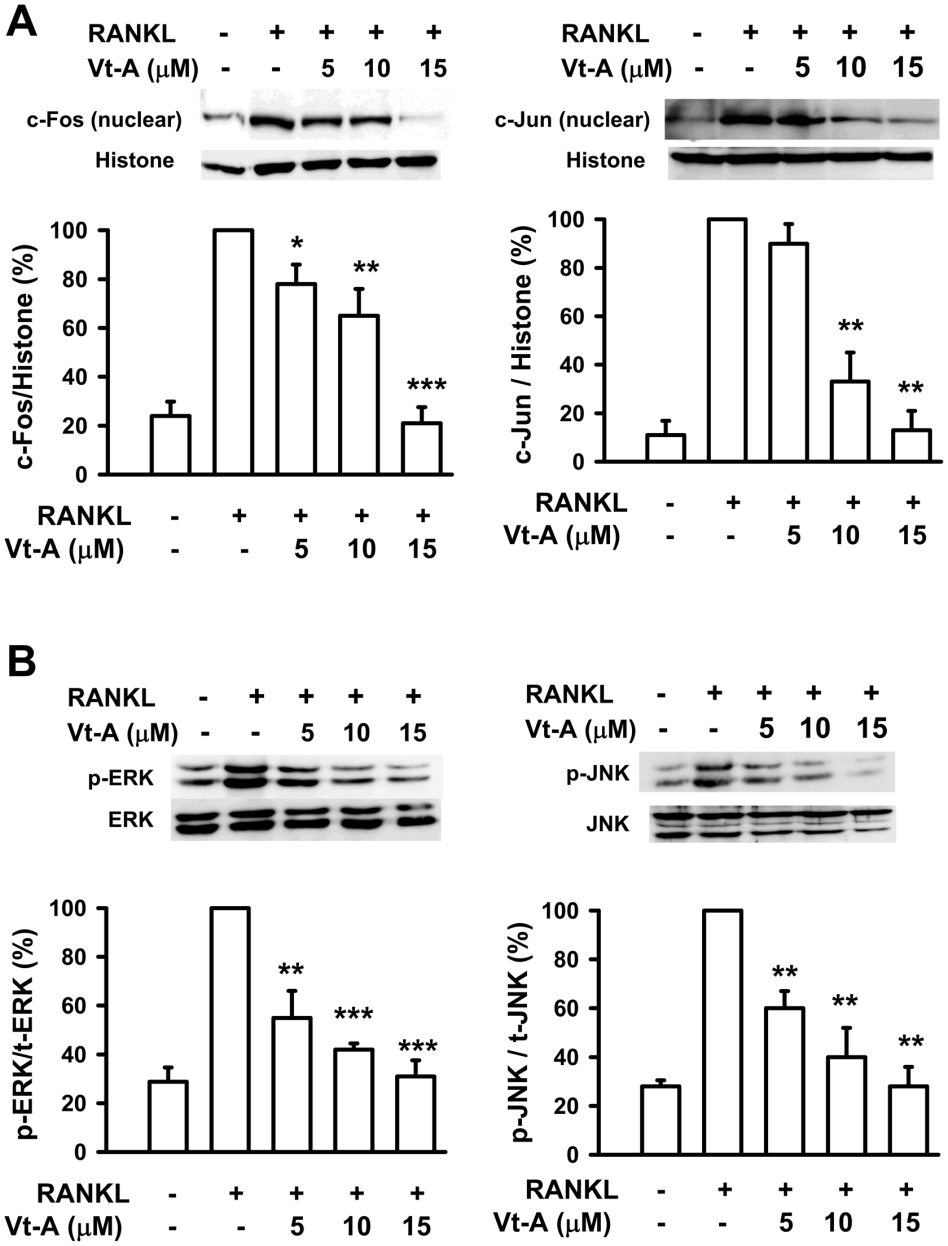
(+)-Vitisin A (Vt-A) suppressed RANKL-induced activation of AP-1. Effects of Vt-A on the (A) nuclear translocation of c-Fos and c-Jun and (B) upstream phosphorylations of ERK and JNK in RAW264.7 cells stimulated with RANKL for 1 day and 15 min, respectively. Activation of signaling molecules were then evaluated by Western blot analysis. Results are expressed as the mean ± SEM for each group from four to five separate experiments normalized for histone (for nuclear protein normalization) or total ERK/JNK (for cytosolic protein normalization) respectively. *p<0.05, **p<0.01 and ***p<0.001, different from values after stimulated with RANKL alone.

Binding of RANKL to RANK activates several transcription factors responsible for promoting osteoclastic gene expression. These are not all activated within the same time frame: early response factors, such as AP-1 (c-Fos/c-Jun dimer), are activated before late-response factors, such as NFATc1 [34]. Furthermore, AP-1 is known to regulate the expression of NFATc1 by binding to the NFATc1 promoter [35]. The results showed that RANKL increased the activation of c-Fos and c-Jun in RAW264.7 cells, represented as increase in nuclear translocation (Figure 7A). In the presence of (+)-vitisin A, RANKL-evoked nuclear translocation of c-Fos and c-Jun were substantial repressed in a concentration-dependent manner. Several reports have indicated that RANKL induces the activation of MAPKs. These kinases, especially ERK and JNK, also participate in c-Fos and c-Jun activation in osteoclast precursors [36], [37]. As shown in Figure 7B, RANKL strongly activated ERK and JNK phosphorylation when measured at the early time point of 15 min and this phosphorylation was inhibited by (+)-vitisin A.

**Figure 8:**
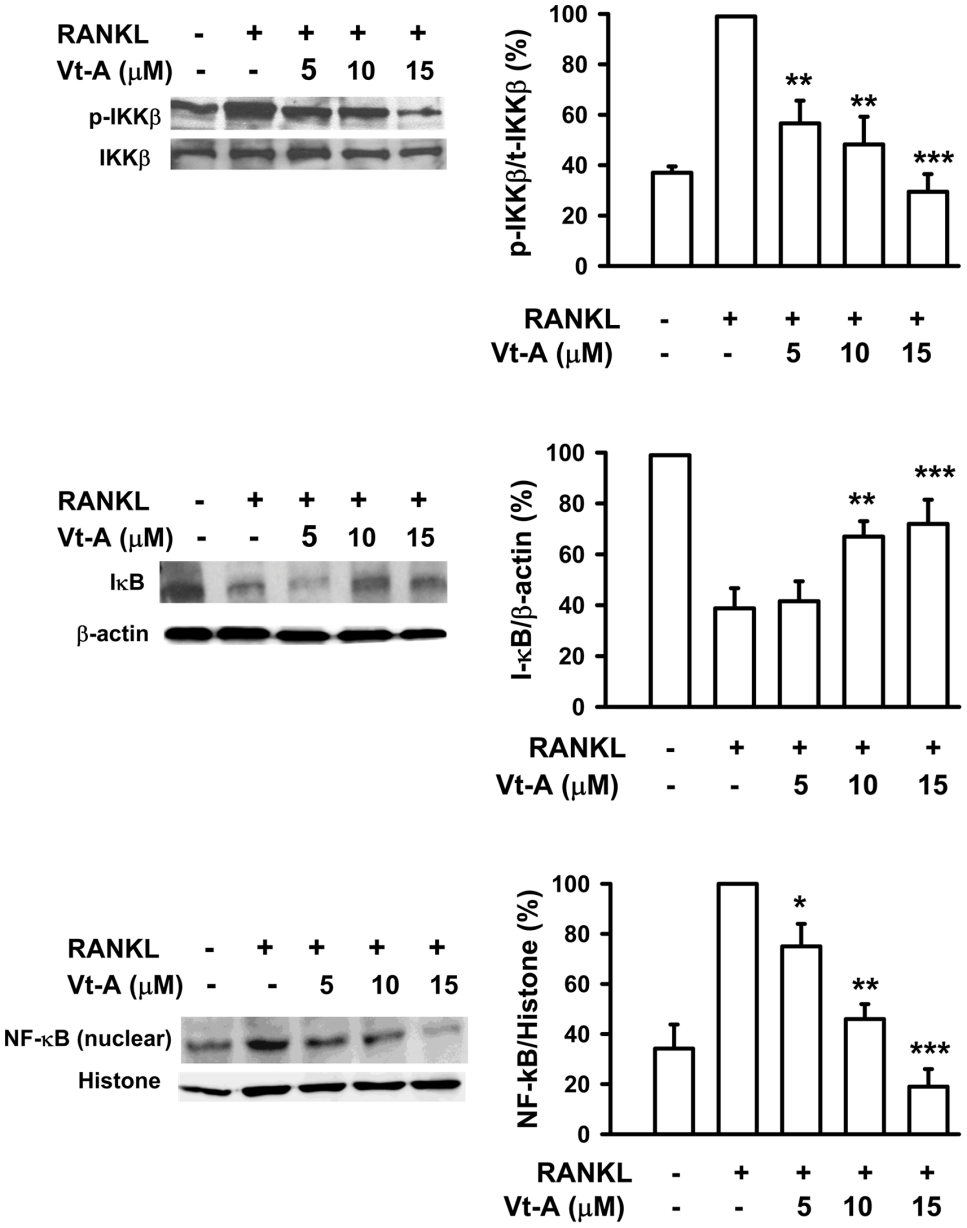
(+)-Vitisin (Vt-A) concentration-dependently repressed RANKL-induced phosphorylation of IKKβ, degradation of IκB and nuclear translocation of NF-κB, respectively. Protein samples were prepared after 15 min (for IKK and IκB) or 1 h (for NF-κB) of RANKL stimulation then analyzed by Western blotting. Results are expressed as the mean ± SEM for each group from four to five separate experiments. Relative protein level was normalized by histone (for NF-κB), total IKK (for phosphorylated IKK) or β-actin (for IκB), respectively. *p<0.05, **p<0.01 and ***p<0.001, different from values after stimulated with RANKL alone.

NF-κB activity is important for RANKL-mediated induction of NFATc1 at the early phase of osteoclastogenesis [38], [39]. Activity of NF-κB is regulated by its inhibitor IκB, which forms a complex with NF-κB in the cytoplasm. After degradation of IκB through an ubiquitin/proteasome pathway, NF-κB is subsequently translocated from the cytoplasm into the nucleus. As shown in Figure 8, RANKL triggered IκB degradation after 15 min incubation, and after 1 h it produced a significant increase in nuclear translocation of NF-κB (65 kD). In the presence of (+)-vitisin A, RANKL-induced IκB degradation and the subsequent nuclear translocation of NF-κB were clearly suppressed. This finding is further supported by Western blot analysis of upstream IKK phosphorylation. As shown in the top of Figure 8, IKKβ phosphorylation was significantly enhanced by RANKL stimulation. When cells were treated with (+)-vitisin A, the RANKL-induced IKKβ phosphorylation was concentration-dependently suppressed. These data firmly suggested that (+)-vitisin A might interfere with the RANKL-activated IκB/NF-κB signaling pathway, and thus contributed to the inhibition of osteoclast formation. However, (+)-vitisin A did not directly affect NF-κB transcriptional activity since DNA binding assay showed a negative result (Figure S1). These data firmly suggested that (+)-vitisin A inhibited osteoclast formation might act through suppressing RANKL-induced IKK/IκB activation rather than interfering with the DNA binding activity of NF-κB.

### RANKL-induced osteoclast differentiation and bone resorption activity from RAW264.7 macrophages is dependent on TRAF6

**Figure 9:**
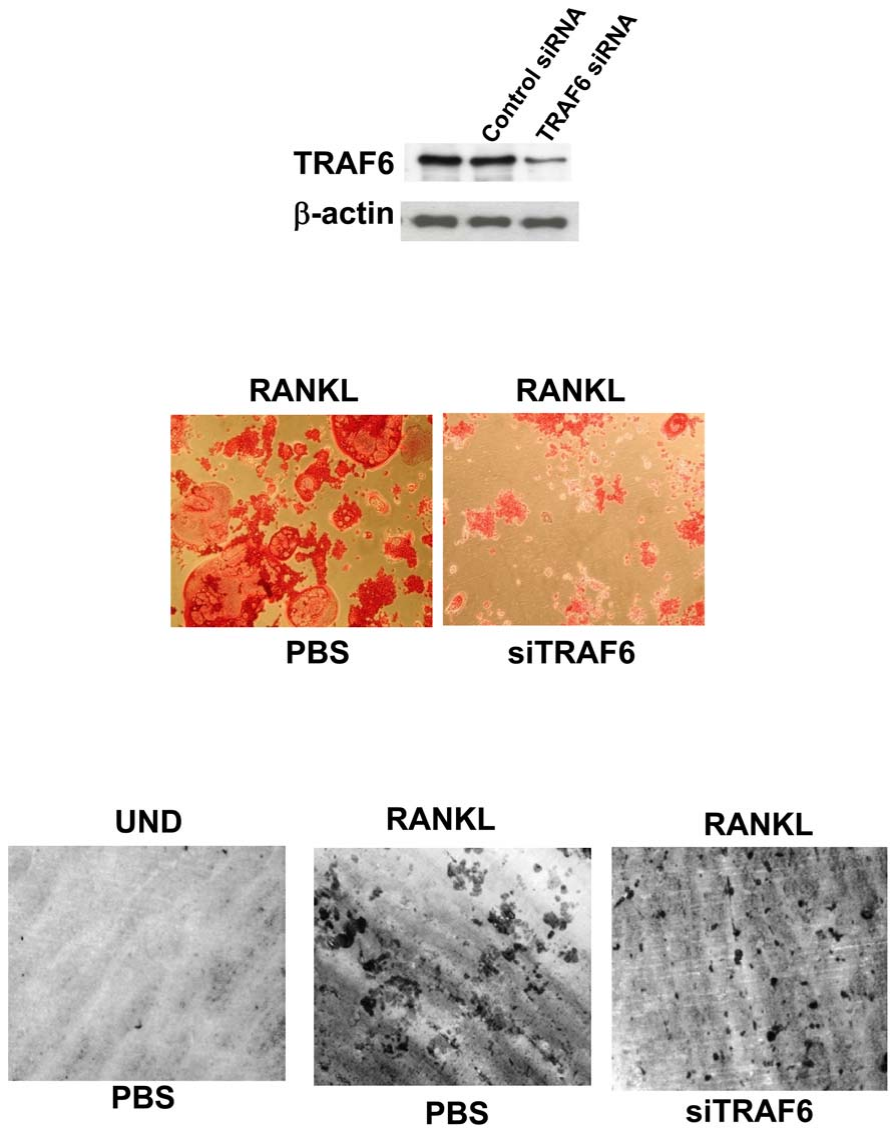
RANKL-induced osteoclast differentiation is dependent on TRAF6. RAW264.7 cells were treated with RANKL in the absence or presence of TRAF6 siRNA or control siRNA as described in methods. TRAF6 expression was analyzed by western blotting before and after siRNA transfection. After incubation, cells were also subjected to analyze TRAP-positive multinuclear cells formation and bone resorption. Cell morphology was examined by light microscopy.

The regulation of osteoclastic signaling is incompletely understood, but ubiquitination of TRAF6 has recently been shown to be important in mediating this process. We therefore investigated whether the TRAF6 signaling pathway is essential for RANKL-induced osteoclast differentiation and bone resorption activity in RAW264.7 cells. As shown in Figure 9, the formation of MNCs (middle) and extensive resorption area (bottom) were noted in cells stimulated with RANKL, however, such differentiation and resoprtion activity were obviously repressed in the presence of siRNA that targeted TRAF6. These results demonstrated that TRAF6 is essential for RANKL-induced osteoclast differentiation and bone resorption activity in RAW264.7 macrophages, as in bone marrow-derived macrophages (BMMs).

### (+)-Vitisin A negatively regulates TRAF6 ubiquitination and subsequent TRAF6-TAK1 association

**Figure 10:**
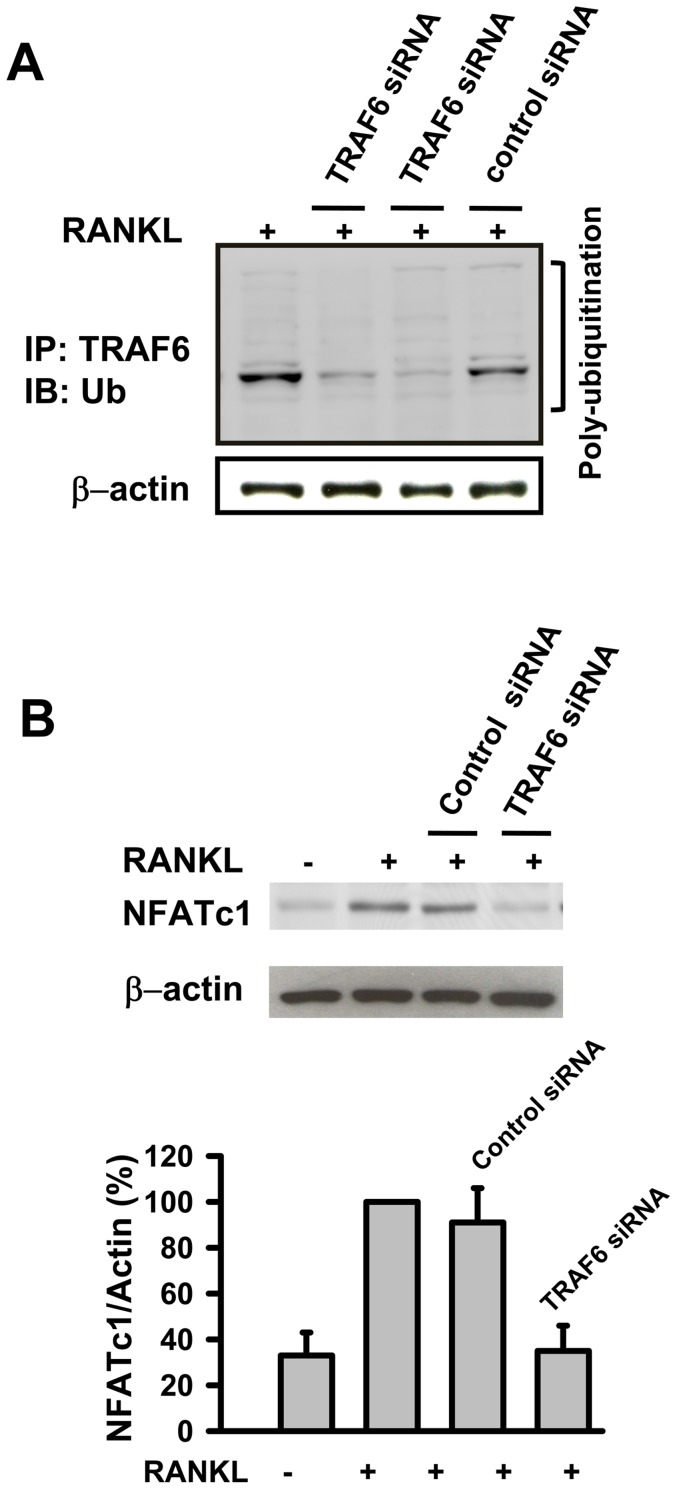
TRAF6 is poly-ubiquitinated after RANKL stimulation and RANKL-induced expression of NFATc1 in osteoclast differentiation is dependent on TRAF6. (A) RAW264.7 cells were stimulated with RANKL in the absence or presence of TRAF6 siRNA (the results represented are two separate experiments) or control siRNA. After incubation, cell lyses were immunoprecipitated with anti-TRAF6 antibody. Bound proteins were further immunoblotted with anti-ubiquitin or anti-β-actin as described in methods. (B) RAW264.7 cells were stimulated with RANKL in the presence or absence of TRAF6 siRNA or control siRNA. After stimulation, cells lysates were immunoblotted with anti-NFATc1 or anti-β-actin antibodies. Results are expressed as the mean ± SEM for each group from three to four separate experiments. **p<0.01, different from values after treatment with RANKL alone.

**Figure 11:**
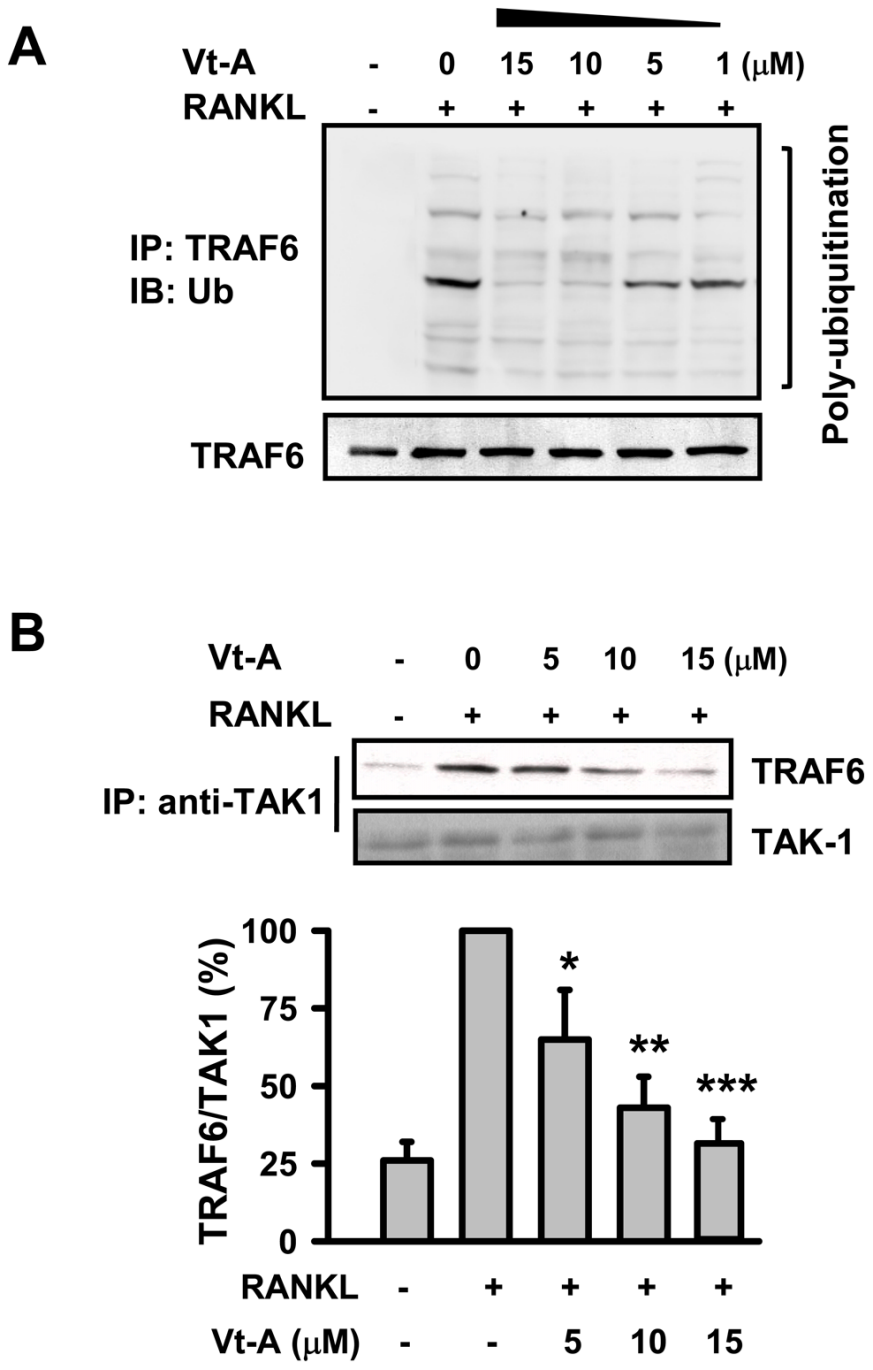
(+)-Vitisin A (Vt-A) suppressed the accumulation of ubiquitinated TRAF6 and the association of TRAF6-TAK1 complex. (A) RAW264.7 cells were stimulated with RANKL in the absence or presence of (+)-vitisin A. After incubation, cell lyses were immunoprecipitated with anti-TRAF6 antibody. Bound proteins were further immunoblotted with anti-ubiquitin or anti-TRAF6 antibody. (B) Cell extracts were immunoprecipitated with anti-TAK1 antibody. Bound proteins were further immunoblotted with anti-TRAF6 or anti-TAK1 antibody. Results are expressed as the mean ± SEM for each group from three to four separate experiments. *p<0.05, **p<0.01 and ***p<0.001, different from values after treatment with RANKL alone.

TRAF6 is a master signaling molecule controlling multiple downstream pathways induced by RANKL. Ubiquitination of TRAF6 plays an important role in its signaling function, including the regulation of osteoclastogenesis [5], [6], [40]. The finding that (+)-vitisin A down-regulated both RANK signaling and osteoclastogenesis prompted us to examine whether this compound altered ubiquitination of TRAF6. As shown in Figure 10A, poly-ubiquitinated TRAF6 was detected in RANKL-stimulated RAW264.7 cells. The ubiquitination is TRAF6-dependent since less ubiquitinated TRAF6 was accumulated in siTRAF6-transfected cells. Moreover, silencing of TRAF6 by siRNA substantially inhibited RANKL-induced induction of NFATc1 in RAW264.7 macrophages (Figure 10B). These results clearly demonstrate that RANKL-induced ubiquitination of TRAF6 is involved in NFATc1 activation and osteoclastogenesis. We next examined the effect of (+)-vitisin A on the accumulation of ubiquitinated TRAF6. As shown in Figure 11A, the expression of TRAF6 was up-regulated when stimulated with RANKL in RAW264.7 macrophages. Significantly, RANKL-mediated TRAF6 ubiquitination was severely impaired in (+)-vitisin A treated cells (Figure 11A). In contrast, (+)-vitisin A failed to affect the total level of TRAF6.

TAK1 is a member of the MAPK kinase kinase (MAPKKK) family with the ability to phosphorylate both MAPK and IKK, so this kinase is considered to be at the branching point of the two pathways. TAK1 is recruited for the formation of TRAF6-TAK1 complexes after stimulation with RANKL in cells. Having identified that the inhibition by (+)-vitisin A is likely to be at the level of TRAF6 ubiquitination, we next analyzed whether (+)-vitisin A could inhibit the downstream formation of the active TRAF6-TAK1 complex. Results showed that RANKL enhanced the association of TRAF6-TAK1 observed at 5 min as previous found [25]. In the presence of (+)-vitisin A (5, 10 and 15 µM), RANKL-induced TRAF6-TAK1 association was concentration-dependently repressed (Figure 11B).

### Effect of (+)-vitisin A on c-Src/β3 association

**Figure 12:**
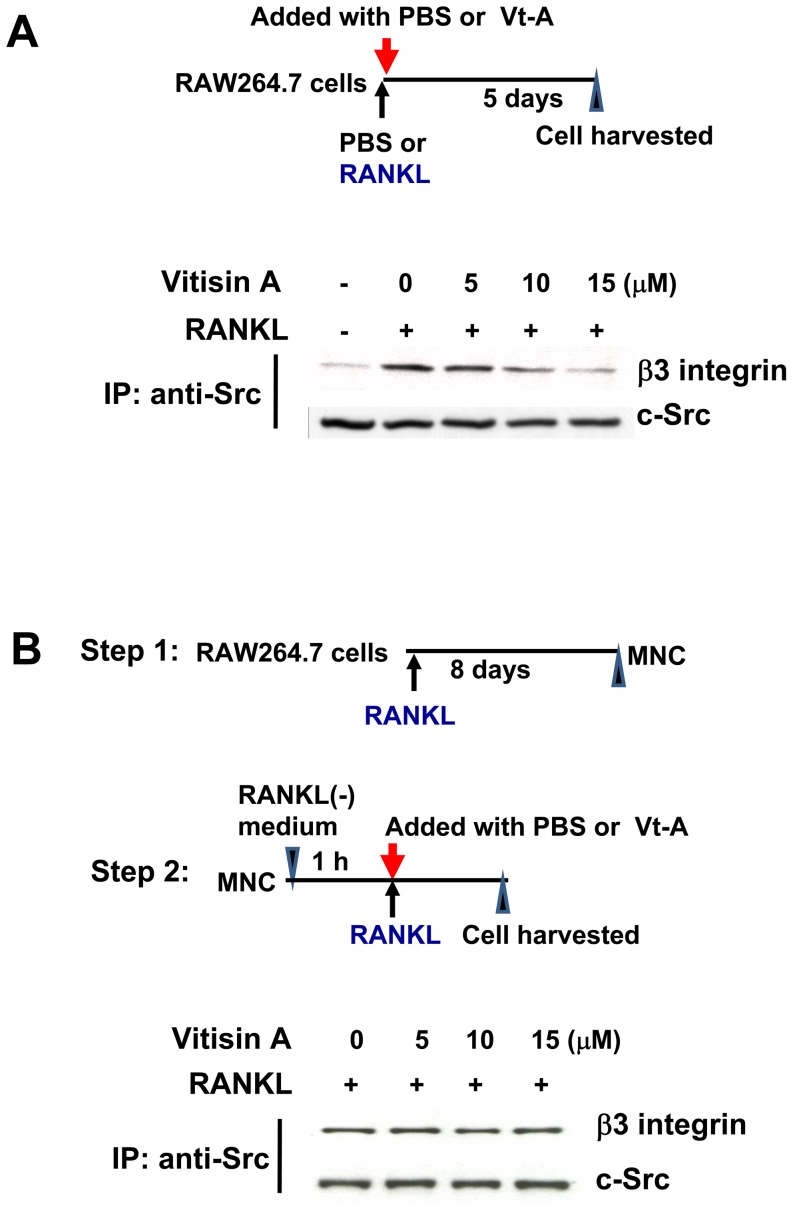
Effect of (+)-vitisin A on c-Src/β3 integrin complex association. (A) RAW264.7 cells were exposed to RANKL for 5 days in the simultaneous presence or absence of (+)-vitisin A. After incubation, cell lyses were immunoprecipitated with c-Src antibody. Bound proteins were subjected to SDS-PAGE and immunoblotted with β3 integrin antibody. The amounts of c-Src in each immune complex were determined by immunoblotting (bottom panel). (B) RAW264.7 cells were exposed to RANKL for 8 days to produce fully differentiated, multinucleated osteoclasts (MNCs). Following 12 h incubation in RANKL-free medium, MNCs were second exposed to RANKL alone or RANKL plus (+)-vitisin A for 30 min. c-Src immunoprecipitates (IP) were immunoblotted for β3 integrin and c-Src, respectively.

Activated RANK prompts two distinct signaling pathways: one promotes osteoclast formation through signaling pathway as above described, and the other, in which the c-src tyrosine kinase (c-Src) and αvβ3 integrin are required, organizes the cell's cytoskeleton and stimulates its resorptive capacity [9]. Furthermore, an interaction of Src with β3 integrin (Src/β3) was observed in RANKL-stimulated cells [41]. These observations prompted us to ask whether interfering with these two molecules association were related to (+)-vitisin A-mediated inhibition of osteoclasts resorption. To explore the c-Src/αvβ3 association in osteoclasts, RAW264.7 cells were exposed to RANKL for 5 days in the absence or presence of (+)-vitisin A. Cell lysates were immunoprecipitated with c-Src then immunoblotted for β3 integrin. Results shown in Figure 12A demonstrated that the formation of c-Src/β3 complex was observed in RANKL-differentiated cell and such phenomenon was obvious repressed when cells were concomitant treated with (+)-vitisin A.

In the other experiment, a two-step design was performed. As shown in Figure 12B, RAW264.7 cells were exposed to RANKL for 8 days to produce multinucleated osteoclasts (MNCs). After that, MNCs were incubated in RANKL-free medium. Following 12 h starvation, mature MNCs were second exposed to RANKL or RANKL plus (+)-vitisin A for 30 min then cells were harvested. In RANKL-starved MNCs, a fainter band of c-Src/β3 complex was observed (data not shown). However, challenged with RANKL obviously induced c-Src/αvβ3 association again. Such interaction was not attenuated by post-treatment with (+)-vitisin A (Figure 12B).

## Discussion

The RANK signaling pathway of osteoclasts provides the molecular basis for developing therapeutics to treat osteoporosis and pathological diseases of bone loss [42]. In this study, we found that (+)-vitisin A significantly inhibits the RANKL-induced osteoclast differentiation of RAW264.7 macrophages, as measured by TRAP activity, multinucleation, and bone resorption by the cells. β3 integrin plays important roles in regulating bone resorption of osteoclasts by mediating their migration and adhesion. Those integrin-deficient mice develop enhanced bone mass as a result of osteoclast dysfunction [43]. OC-STAMP is the key fusion-mediating molecule and its expression regulates osteoclast multinucleation. In OC-STAMP knock-out mice, the complete abrogation of osteoclast cell fusion and foreign body giant cell formation by macrophage suggested its role in cell fusion [44]. The results presented here indicated a markedly induction of β3 integrin and OC-STAMP expression in RANKL-stimulated RAW264.7cells and such responsiveness was potently inhibited by (+)-vitisin A. Several proteolytic enzymes, including TRAP, cathepsin K, MMP-13 and MMP-9, have been demonstrated to play important roles in degrading the organic bone matrix. Of these, cathepsin K and MMP-9 have the highest levels in osteoclasts. Both matrix-degrading enzymes are known to be collagen-degrading enzymes, and they can directly degrade collagens in hard tissues that have been demineralized [19]. In this study, stimulation with RANKL resulted in the formation of many resorption pits on bone slices, and (+)-vitisin A treatments dramatically diminished pit areas. Results obtained from Western blotting, gel zymography and spectroflurometric assay further revealed that the anti-bone resorption effect of (+)-vitisin A attributes to not only the decreased protein levels of cathepsin K and MMP-9, but also the diminished enzyme activity.

**Figure 13:**
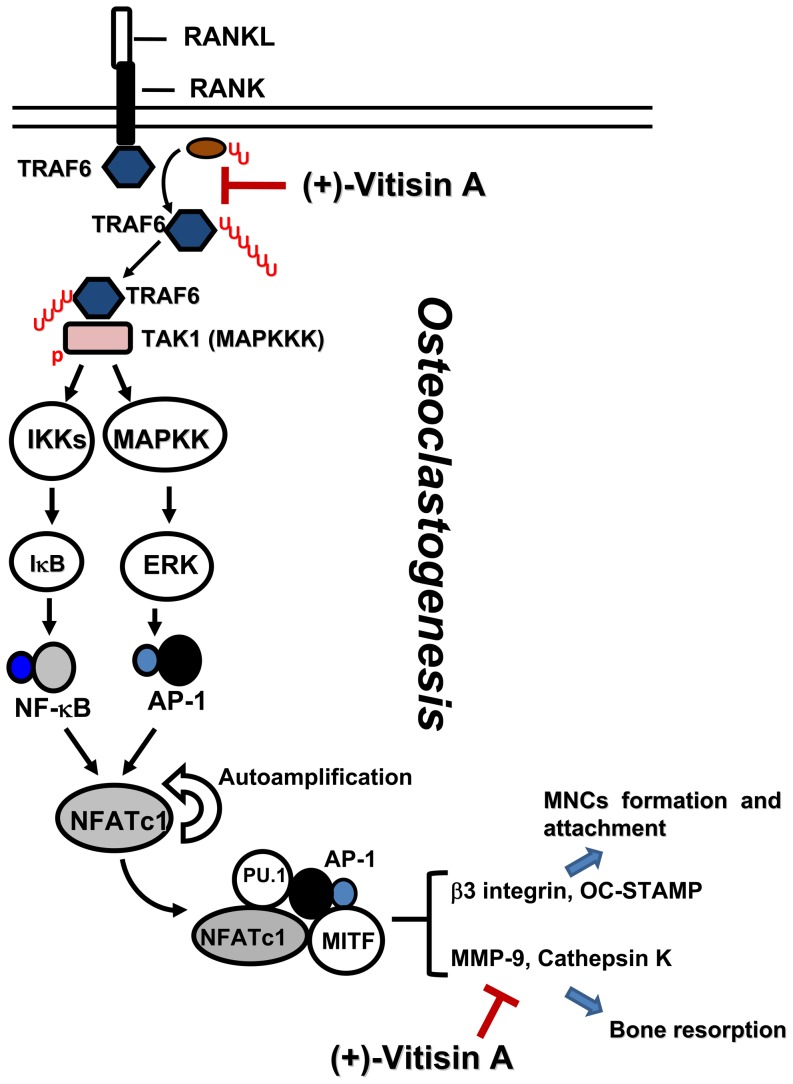
Proposed intracellular mechanisms of (+)-vitisin A in suppressing RANKL-induced osteoclastogenesis in RAW264.7 cells. The underlying mechanisms involved interfering with the ubiquitination of TRAF6 and downstream signaling cascades to suppression the expression of osteoclast marker proteins and bone resorption. Furthermore, down-regulation of matrix-degrading enzymes activity (cathepsin K and MMP-9) by (+)-vitisin A might also contribute to the beneficial effect on preventing bone loss. AP-1, activator protein 1; DC-STAMP, dendritic cell-specific transmembrane protein; MAPKs, mitogen-activated protein kinases; MITF, microphthalmia-associated transcription factor; MMP-9, matrix metalloproteinase-9; NFATc1, nuclear factor of activated T cells c1; RANKL, receptor activator of nuclear factor κB (NF-κB) ligand; TAK1, transforming growth factor β-activated kinase (TAK)-1; TRAF6, tumour necrosis factor receptor-associated factor 6.

The present molecular pathways underlying osteoclast formation are illustrated in Figure 13 which included the sequential molecular events induced by RANK [45]. The binding of RANKL to RANK results in the recruitment of TRAF6-TAK1, which activates NF-κB and MAPKs. NF-κB is important for the initial induction of NFATc1. NFATc1 binds to its own promoter, thus switching on an autoregulatory loop [46]. The induction of NFATc1 is also dependent on the MAPKs-mediated activation of AP-1 complex (c-Fos/c-Jun dimer) and this complex is required for the further auto-amplification of NFATc1, enabling the robust induction of NFATc1 [47]. Finally, NFATc1 cooperates with other transcriptional partners such as NF-κB, MITF and PU-1 to activate osteoclast-specific genes [48]-[50]. It has been reported that NFATc1-deficient embryonic stem cells fail to differentiate into mature osteoclasts in response to RANKL [11]. In addition, exogenous overexpression of NFATc1 in BMMs efficiently induces differentiation of these cells into osteoclasts even in the absence of RANKL [11], [51]. Our results revealed that RANKL markedly up-regulated the expressions and nuclear translocations of both c-Fos/c-Jun and NFATc1, and that (+)-vitisin A treatment not only suppressed the expression of c-Fos/c-Jun and NFATc1, but also dramatically suppressed the nuclear translocations of these transcription factors, as evidenced by their reduced amounts in the nucleus. Thus, we suggested that the inhibition of RANKL-induced AP-1 activation by (+)-vitisin A is a relevant factor in the suppression of downstream NFATc1 signal pathways. Activation of the above-mentioned signaling pathways directly regulates a number of osteoclastogenesis-related markers, including OC-STAMP, integrin β3, MMP-9 and cathepsin K, which lead to the formation of bone resorption pits during osteoclast differentiation [52]. Thus, the prevention of RANKL-induced bone resorption by (+)-vitisin A may be, at least in part, attributable to down-regulating NFATc1 expression via targeting of AP-1. In addition, MAPKs have been implicated in RANK signaling by regulation of AP-1 activation. Our results showed that (+)-vitisin A significantly inhibits RANKL-induced phosphorylations of ERK and JNK. A reasonable explanation for this might be that (+)-vitisin A suppressed AP-1 activation via the inhibition of upstream MAPKs activation. The importance of NF-κB in RANK signaling pathways for osteoclastogenesis has been confirmed by genetic studies [13], [53]. The present results indicated that (+)-vitisin A attenuated not only RANKL-induced IKK phosphorylation, but also the subsequent degradation of IκB and nuclear translocation of NF-κB, suggesting that the inhibitory effect of (+)-vitisin A on osteoclast differentiation might also dependent on the NF-κB activation pathway.

As a multifunctional second messenger activated by RANKL, TRAF6 is critical for RANK-induced activation of downstream signal factors such as NFATc1. In RANKL signaling, the cytoplasmic domain of RANK recruits TRAF6 to initiate a signaling cascade that is crucial for the maturation of monocyte precursors to fully differentiated osteoclasts. Our results supported the critical role of TRAF6 in RANK signaling and osteoclast maturation in RAW264.7 cells, as evidenced by NFATc1 expression and bone resorption were seriously repressed when TRAF6 was silenced by transfected with siRNA. It has been shown that TRAF6 forms a signaling complex containing RANK and TAK1-binding protein (TAB)2, resulting in TAK1 activation [8], and subsequent activation of NF-κB, AP-1 and p38 pathways, which are crucial for osteoclast differentiation, survival and function [53]. These results are observed in TRAF6-deficient mice which develop osteopetrosis due to reduced osteoclasts [54]. To clarify the roles of TRAF6 and TAK1 in our model, RAW264.7 cells were stimulated with RANKL and the formation of TRAF6-TAK1 complexes was measured. Based on our immunoprecipitation observations, stimulation with RANKL indeed evoked a rapid and significant accumulation of TRAF6-TAK1 protein complexes, and the amount of such complexes was less in the presence of (+)-vitisin A. The data presented here demonstrate that (+)-vitisin A might disrupt intracellular signal propagation by targeting the formation of upstream TRAF6-TAK1 complexes.

TRAF6, as a unique member of a family of RING domain ubiquitin ligases, can catalyze polyubiquitin chains linked through Lys-63 of ubiquitin [40], [55]. Lamothe et al. [40] identified that site-specific TRAF6 auto-ubiquitination is prerequisite to initiate a cascade of further downstream ubiquitination events, including the ubiquitination of components in the TAK1 and IKK complexes. In addition, TAK1 is recruited for formation of the TRAF6-TAK1 complex after stimulation with RANKL in cells. This complex thereafter activated MAPKs and NF-κB pathways. Based on the findings obtained from western blotting and immunoprecipitation, RANKL stimulation indeed evoked the ubiquitination of TRAF6 and the accumulation of TRAF6-TAK1 complexes in RAW264.7 cells. Results showed that (+)-vitisin A treatment had no effect on the expression of TRAF6, but significantly suppressed the ubiquitination of TRAF6 and subsequent accumulation of TRAF6-TAK1 complexes. Further studies are required to explore how (+)-vitisin A interferes in poly-Ub chain ligation.

The interaction of c-Src and integrin initiates signaling that leads to the complex intracellular cytoskeletal reorganization that is required for polarization of the cytoplasm and ruffled border formation. Our results showed the association between c-Src and β3 integrin was markedly induced by RANKL stimulation in RAW264.7 cells. Since (+)-vitisin A strongly down-regulated RANKL-induced β3 expression as shown in Figure 4, results suggested that inhibition of c-Src/β3 interaction by (+)-vitisin A attributed to the decreased level of β3 and established that the c-Src/β3 complex interacted in a β3 integrin-dependent manner. However, (+)-vitisin A failed to affect the assembly of c-Src/β3 complex when post-exposure to MNCs.

Histone deacetylases (HDACs) are named for their deacetylase activity toward lysine residues in histones; however, it is important to recognize that they also deacetylate other proteins and they predate histones in evolution [56]. HDACs have crucial roles in organizing the actin cytoskeleton and microtubule network, which is extremely important during osteoclasts recruitment to remodeling sites, formation of the sealing zone, and bone resorption. Several natural and synthetic small molecule inhibitors of HDACs exist (such as trichostatin A, depsipeptide, SAHA, sodium butyrate or MS-275) [57]. Current in vitro evidence indicates that inhibiting HDACs promotes osteoblast maturation and suppresses osteoclast maturation. HDAC inhibitors (HDIs) accelerate alkaline phosphatase production and matrix mineralization of osseous cells in vitro and calvarial explants ex vivo [58], [59]. In contrast to their positive effects on in vitro osteoblast maturation, HDIs decrease the survival and maturation of osteoclasts [60]. For example, trichostatin A suppressed the differentiation of osteoclasts from bone marrow cultures [61] and induced p21WAF expression, which contributed to osteoclast apoptosis [62]. Depsipeptide suppressed in vitro osteoclastogenesis by blocking RANKL-induced nuclear translocation of NFATc1 and by increasing production of IFN-γ, an inhibitor of osteoclastogenesis [63]. On the other hand, SAHA abolished osteoclastogenesis by suppressing several events leading to NF-κB activation [64]. Together with the extensive literature documenting the anti-osteoclastogenetic effects of HDIs, these data suggest that targeting HDACs might be a novel strategy for treating diseases associated with abnormal bone mass and strength. Whether (+)-vitisin A acts as a HDI to prevent osteoclast maturation needed further study.

In conclusion, the results suggested that impeding the differentiation and function of osteoclasts might contribute to the anti-resorption activity of (+)-vitisin A. The underlying mechanisms involved interfering with the ubiquitination of TRAF6, the accumulation of TRAF6-TAK1 complexes and the activation of IKK/NF-κB/c-Fos/NFATc1 cascades (Figure 13). Inhibition of these signaling pathways finally led to the suppression of expression of osteoclast marker proteins. Furthermore, down-regulation of matrix-degrading enzymes activity (cathepsin K and MMP-9) by (+)-vitisin A might also contribute to the beneficial effect of VtR (a (+)-vitisinA-enriched preparation from *Vitis thunbergii*) in preventing estrogen deficiency mediated bone lose in ovariectomized mice.

## Supporting Information

Figure S1 **(+)-Vitisin A (Vt-A) did not directly affect NF-κB transcriptional activity.** Nuclear extract was obtained from RAW 264.7 cells stimulated with RANKL alone, then (+)-vitisin A was post-added to the reaction mixture including nuclear extract and DNA probe. The NF-κB transcriptional activity was performed by using a commercial NF-κB (p65) Transcription Factor Assay Kit (Cayman Chemical, Ann Arbor, MI). Results are expressed as the mean ± SEM for each group from three to four separate experiments.
(TIF)
