# Supplementary figures and images for: (+)-Vitisin A Inhibits Osteoclast Differentiation by Preventing TRAF6 Ubiquitination and TRAF6-TAK1 Formation to Suppress NFATc1 Activation
Source: PLoS One. 2014 Feb 18;9(2):e89159. doi: 10.1371/journal.pone.0089159 (PMC3928435; doi:10.1371/journal.pone.0089159)

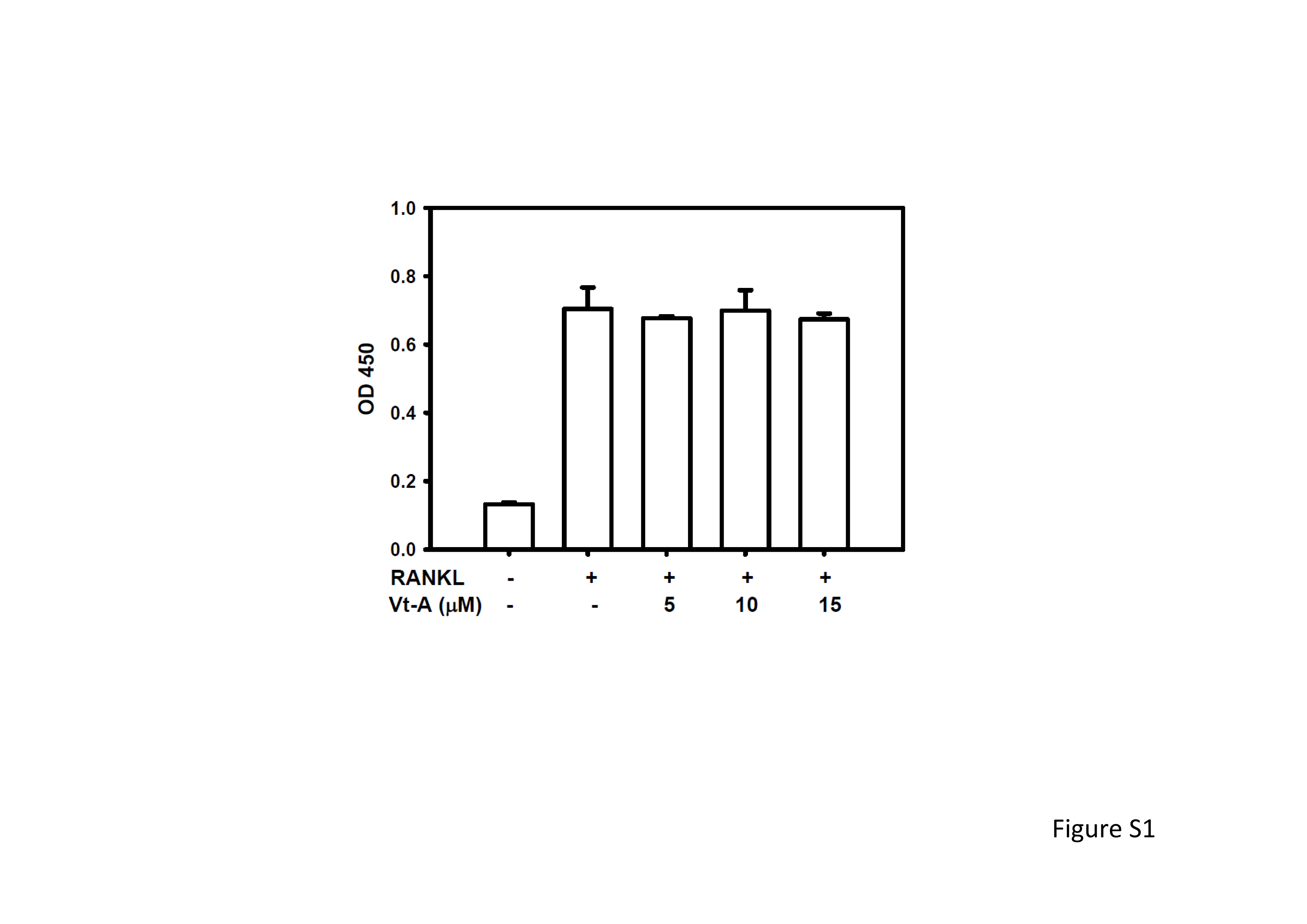

Supplement: Figure S1 — (+)-Vitisin A (Vt-A) did not directly affect NF-κB transcriptional activity. Nuclear extract was obtained from RAW 264.7 cells stimulated with RANKL alone, then (+)-vitisin A was post-added to the reaction mixture including nuclear extract and DNA probe. The NF-κB transcriptional activity was performed by using a commercial NF-κB (p65) Transcription Factor Assay Kit (Cayman Chemical, Ann Arbor, MI). Results are expressed as the mean ± SEM for each group from three to four separate experiments. (TIF) [file pone.0089159.s001.tif]
